# Bridge Over Troubled Water: Shared Understanding Bridges Individual and Collective Resources in Developing Team Resilience in Professional Football

**DOI:** 10.3389/fspor.2021.705945

**Published:** 2021-12-01

**Authors:** Ole Erik Grinde

**Affiliations:** Department of Teacher Education, Faculty of Social Science and Education, Norwegian University of Science and Technology, Trondheim, Norway

**Keywords:** coordination, interaction, team identity, member knowledge, collaboration

## Abstract

This study explored how coaches facilitate coordinated activities through shared understanding in the processes of team resilience development. Constructs of shared information that underpin synchronised actions and behaviour in a team are investigated through individual experiences with a dialogic “we” perspective of appropriating and handling challenging situations. Interactional key elements underpin coordinated task actions within the team. Experiences of both players and coaches are investigated through semi-structured interviews and complementary texts such as an observation log and coach-meeting reports, originating as part of an action research process in the team environment. The interaction model is developed in the exploratory journey during the season with the team. The model suggests key strategic elements that help to bridge shared appropriation of information to strengthen role interactions between team members handling challenging situations. Coaching practise, which connects the interaction model to different team resources of coordinating activities in the development process, still needs to be explored from different contextual perspectives and environments, within the development of team resilience.

## Introduction

The development of team resilience, through mobilising collective resources, is promoted as a key to sporting success (Decroos et al., 2017).

Characteristics of team resilience are established, and different aspects are revealed in sports teams, but less is known about the development process (Kegelaers et al., 2020). The development of team resilience is recognised as an adaptive and dynamic process, coming from an individual, contextual and team-level interactions, where leadership has a key role in such development (Bowers et al., 2017; Gucciardi et al., 2018). Teams should avoid, what Wergin et al. (2018) describe as the collective collapse in critical events, which have negative impacts on the affective, cognitive, and behavioural responses within the team.

To manage the development of needed positive responses, the role of coaches is highlighted for integrating the development process into daily practise (Fletcher and Sarkar, 2016) through transformational and shared leadership (Morgan et al., 2017). Coaches being strong leaders together with shared leadership are shown to be valuable in dealing with highly stressful conditions while competing in international arenas (Kegelaers et al., 2020).

In the development of team resilience, Decroos et al. (2017) has argued that teams need to avoid vulnerabilities such as negative communication and lack of effort. The teams need to establish ownership within the group, in order to strengthen communication channels (Kegelaers et al., 2020). Developing such communication channels through closed-loop communication and mutual trust, cultivated by enablers such as shared vision, encouragement of honest feedback, and distributed responsibility, has been found to improve the coordinating mechanisms of the team during difficult periods (Morgan et al., 2019).

To develop a team-regulatory system, teams should give members the responsibility for the functioning and working communication (both verbal and non-verbal) in order to handle challenging situations by being, “on the same wavelength.” In Morgan et al.'s (2019) work, it is recognised through statements like, “we knew what to do,” and, “nothing was a surprise.” The utilisation of regular exposure to challenging situations, as well as establishing team briefings to openly discuss how the teams function, seem to develop coordinative mechanisms and quality interactions.

The importance of such quality interactions is promoted by the investigation of Kegelaers (2019) on the role of coaches in the development of team resilience. His work pinpoints the need for coaches to develop interpersonal skills without digging deeper into quality aspects of communication and complex aspects of interaction (Kegelaers, 2019). The work of Morgan et al. (2019) presents the need of establishing protocols for handling high-pressure situations, in order to establish specific actions and cues within the team. Their development is recommended through team characteristics, coordinative, and communicational guidelines, without the authors digging deeper into how the knowledge of team members might influence the interacting processes itself.

While exploring coping strategies LePrince et al. (2018) promote that little attention is paid to the collective efforts and cooperative actions developed by teammates to withstand team-specific stressors. Developing specific coping strategies for dealing with communal sources of stress is suggested by LePrince et al. (2018). This is in line with both the recommendation of Morgan et al. (2019) of establishing team-regulatory systems and the suggestion of Kegelears et al. (2020) for both players and coaches to take ownership of strengthening communication channels within handling challenging situations.

To the knowledge of the author, the communications aspect of such coordinated handling of challenges has yet to be investigated in professional football, and how to facilitate the appropriation of team members of shared understanding needs further exploration. To make common sense of the situation is essential, and in daily practise the implementation of proactive or reactive coordinated actions to deal with challenges within the team is essential. Incorporating such practise during planning or debriefing set-ups from day-to-day activities is part of the responsibilities of coaches. The implementation of resistance practise with team members involves being “on the same wavelength” (Morgan et al., 2019) and improves the quality of interaction within the team (Kegelaers, 2019).

The suggested coordinative mechanisms handling challenging situations need to be further investigated to be able to facilitate context-specific team protocols in the team resilience development process. To be able to establish interpersonal links both implicit and explicit through strengthening communicational channels, knowledge on processes might improve the interaction between team members. Such Knowledge from studies on music experts, related to the characteristics of shared experiences (Szanto and Krueger, 2019) in the sense of developing plural self-knowledge through an intentionally shared agency, has transferred value for coaches in facilitating a framework for the development of joint collaborative actions.

Studying the collaborative interaction of musicians at a world-class level, Salice et al. (2019) suggest musicians develop a consciousness based on a “we-narrative” of “motor-resonance,” “explicit coordination,” and “interkinesthetic affectivity.” The sense of shared agency is connected to and strongly dependent on, triggers within the cognitive and affective process (Salice et al., 2019). The interaction between individuals in a joint activity unfolds as both symmetrical and complementary relations of co-workers.

A depiction of the “we-narrative” which Salice et al. (2019) connect to interkinesthetic affectivity and felt trust, characterised by communicated narratives. These reflections define the understanding of the group about their past, present, and future actions as well as their intentions, goals, and norms (Szanto and Krueger, 2019). In the understanding of the author, the “we-narrative” Szanto and Krueger (2019) present, needs a dialogic frame (Rommetveit, 2008). This helps players collectively handle challenging situations. In this way team members (players and coaches) practise developing a shared understanding of the challenging situation by letting it make sense within a “we” perspective.

Investigating work teams, Talat and Riaz (2020), claimed team sensemaking has a significant impact on team resilience. This implies that teams manage and coordinate effort through explaining current situations and anticipating future situations in ambiguous and uncertain conditions. They suggest utilising team member bricolage as a combination of resources at hand in the team while developing resilience in a work context (Talat and Riaz, 2020). In professional football, players are expected to fulfil competence in roles and develop behavioural actions coordinated with interdependent team members (Gucciardi et al., 2018).

To be able to synchronise actions and handle task-specific challenging situations together, there is a need to practise cognitive restructuring and information sharing during the process (Bowers et al., 2017). Such coordinative processes require players to have knowledge of the conceptual guidelines of a team, know how other team members operate and know the functions of each other. The development of team member knowledge is suggested by Giske et al. (2018) as part of an implicit learning process, divided into individual strengths and weaknesses, preferences, and abilities of prediction within the role of the player. Its establishment is recommended through role expectations and interaction integrated into the environment (Giske et al., 2018). In this study, team member knowledge is also expected to be experienced through a dialogic (Rommetveit, 2008) frame of practise and interaction.

The facilitation of team members being on “the same wavelength” in developing a shared understanding of handling challenging situations is unexplored in Norwegian professional football. Following a team during a season in the top league (Eliteserien), communicational elements that nurture shared understanding for building resourceful coordinated actions to develop team resilience are investigated. The question guiding this study was: *What strategic elements facilitate shared understanding for handling challenging situations while developing team resilience in professional football?*

## Methodology

This study explored the experiences and reflections of players and coaches when it comes to being challenged and supported within their role in the development processes of the team. It is a qualitative study exploring how shared understanding of important elements in working communication (Morgan et al., 2019) and the strengthening of communication channels (Kegelaers et al., 2020) contribute to developing team resilience within a Norwegian professional football team.

This research emerged through a collaboration between the coaching staff and a researcher with the aim of enhancing team performance. An action research approach is used to reflect and improve own practise within the team (McNiff and Whitehead, 2010; Savin-Baden and Howell-Major, 2013). It was part of a shared initiative to facilitate the development of team resilience through integrated practise during a season in the Norwegian top division (Eliteserien). Activities were systematically implemented to improve practise by changing it through collaboration and reflections within the team and coaches involved in the research.

The approach builds on the assumption of participatory action research generating knowledge with the participants to inform action in a context-specific environment (Savin-Baden and Howell-Major, 2013). Such processes within professional environments (McNiff, 2002), recognise that shared understanding and negotiated participation, are central to the method (Savin-Baden and Howell-Major, 2013). The methodological approach was understood through the lens of participatory action research.

### Organised Activities

Actions were facilitated by transformational and shared leadership and had three different steps, which were, to some extent, integrated. Three steps were initiated to organise group structures in the team with the aim of enhancing working communication and interaction between members through guidelines and role expectations within the identity of the team. All three steps were designed with the intention of enhancing feedback-loops of challenge and support during the season.

First, the transformational leadership approach of coaches has utilised small-sized groups in organisational structures down to the size of dyads in briefing and debriefing performance in challenging situations. The dyadic constructs (e.g., player-player or player-coach) framed cognitive restructuring and information sharing while practising feedback between team members. Feedback was given by systematically asking the questions: *what was good* and *what needed to be improved* during the debriefing using video analyses of crunch-time situations in handling challenging situations. Together with the reflective video analyses, small units were also utilised during pitch practise. These sessions integrated planning, walk-throughs, and adjustments of the strategic game plans of the team. The organised group structures were experimentally designed to enhance the quality of information between players depending on each other in sharing challenging situations.

Second, to reinforce such group processes of players being on “the same wavelength” during practise and competition, the shared leadership group initiated and developed a set of values together with the rest of the players. The process emerged with being *loyal*, giving *100%* regardless of the situation, being *committed, daring* to try, and staying *positive*. The five values were posted on the wall in the locker room as a reminder framing the activities of the team. During the development process in pre-season, the ownership process was also nurtured within the team by letting different small-sized team units discuss and write down descriptions of how the qualities of values were supposed to be shown in referential team activities.

The values were supported by other principal guidelines with coaches expecting players to contribute to the team. Expectations were designed as slogans such as *no one beats the team in work effort*. The slogans were repeatedly presented to coaches and players as visual triggers at every briefing and debriefing presentation. The team identity also founded the description “*we*” are bigger than “*I*” as another guideline for members of the team.

These team characteristics represent elements within the team identity of building a “selfless culture,” which became part of the communication structures facilitated by the shared leadership group as part of the internal justice-regulating mechanisms influencing coordinating activities. The transformational leadership of coaches emphasised actions within these distinctions of feedback while moderating group norms to influence cognitive, affective, and behavioural coordination within the team to handle adverse situations.

Third, conceptualisation on pitch guidelines in line with the identity has provided directions to coordinate collaboration between team members in the development processes. *Expectations* were communicated to claim ownership of interactional *roles* in the team. The demands of roles and activities were structured into the categories of offence and defence positioning play together with skills development. Choices connected with different aspects of the game were collaborated with practising preparedness to face challenging situations with shared understanding and coordinated actions.

To facilitate ownership in the role development process, structures of goal-setting processes and self-regulated learning were utilised in the planning, monitoring, and evaluation of practise and matches. It involved the self-monitoring effort of players and coaches monitoring the quality and quantity of physical movements. The video of players has analysed the role performance together with coaches. Moreover, players self-reported their readiness to perform and rate their perceived exertion and quality of performance every day both during practise and playing matches. The intention of these organised structures was to help players plan, subjectively monitor, and reflect on the content of practise handling challenging situations.

### Data Collection

#### Data Sampling and Participants

Semi-structural interviews (Kvale and Brinkmann, 2010) were conducted with coaches and players in their own environment to gather a broad spectrum of information capturing diverse aspects of individual approaches in a complex development environment like professional football. During the interview, players, and coaches were asked how they dealt with challenging situations.

Follow-up questions were asked for exploring the individual reflections, as recommended by Kvale and Brinkmann (2010), of the players and coaches who utilised these strategies. We reflected on the results of both the successful and unsuccessful handling of challenging situations. It provided the possibility to examine strategic interactional elements during the exploratory process in the team. To investigate strategic elements further constructing the suggested interaction model in this study, interviews were also conducted with elite players and coaches from other environments to obtain referential insight into coordinative actions between players within dyadic constellations in professional football.

To meet the rigorous demands of this methodological approach (Smith and McGannon, 2018), early in the process, the author produced a reflexivity journal about the assumptions and experiences based on current knowledge of the research content. It was done to be aware of the subjectivity of the researcher as recommended by Schinke and Blodgett (2016) in community-based participatory research. During the action and data gathering period, researcher reflections were noted in an observational log and team reports of coaching staff meetings were written.

The written material became supplementary information to the semi-structured interviews in line with the suggestions of Smith and Sparks (2018). Reflections on the material were also discussed with a colleague functioning as a critical friend (Smith and McGannon, 2018) and recorded after the season. This was added to the bank of resources on which this investigation is built. It was utilised to reflect on the positioning of myself as a researcher, together with the reflections of the different coaches and players regarding their experiences.

#### The Role of the Researcher

The researcher was a member of the coaching team when the fieldwork was started.

As part of the coaching staff, relationships were established with players and other coaches. This included the role of being a participatory action researcher throughout the process (Savin-Baden and Howell-Major, 2013). The main responsibility was helping players establish cognitive, emotional, and behavioural baselines to develop the ability to handle challenges. The role also included helping coaching staff interact with players through planning, acting, and debriefing activities by improving working communication in coordinating actions for handling challenging situations. The researcher helped facilitate internal interaction processes within the team, participating in the shared leadership group. All these activities were initiated to develop knowledge within the community to develop and offer solutions on a local level as suggested in participatory action research by Schinke and Blodgett (2016).

Being involved in the team processes, the risk of observational limitations through selective attention, memory, or data entry arose (Cohen et al., 2011). On the other hand, the position in the team made it possible to approach cases from different perspectives by gathering a variety of empirical materials (Smith and Sparks, 2018). The combined role as both a coach and participatory action researcher within the community (Schinke and Blodgett, 2016) following the team for a whole season made the continuous in-depth study possible.

The combination of a written log, team meeting reports, interviews of coaches, players, and the shared leadership group, presented different experiences with both personal and temporal diversity. This strengthens the study by adding multiple truths, perspectives, and results to the research process (Smith and McGannon, 2018).

#### Ethics

The research project was approved by the Norwegian Centre for Research Data (NSD ref. 43712/3/AMS). Members were informed of the study and the mixed role of the researcher. Prior to the study, informed consent was given.

In this study, gaining trust with the team members was a crucial part of the data collection process. Balancing being a member of the coaching staff and functioning as a researcher was a challenging task, especially in handling information in a nuanced and credible way. The process needed to be transparent for both the players and other coaches. The expectation of the researcher was not to be involved in picking the team for a match during the whole season. This was also done to avoid becoming involved in comparing players and other subjective decision-making about the position of the player within the team, which he complied with as part of his role in the environment.

#### Data Analysis

The transcribed material was analysed to code, categorise, and compare the experience of players and coaches (Fejes and Thornberg, 2015). Initially, interviews were coded line-by-line, followed by categories of strategically important elements coming from players and coaches which influence shared understanding of interpersonal actions handling challenging situations within the team. The categories emerged by comparing different experiences (Charmaz, 2014) of both players and coaches.

Information in the interviews was systematically reduced to categories that involved both the player and coaches or multiple players (two or more) in described interaction experiences. The data material consisted of 13 semi-structured interviews from the team, two interviews with both a coach and a player together with one group interview outside the environment. In total, 16 constructed interviews contributed to the study. With the addition of the analysed research log and team meeting references, there was a broad spectrum of material covering the breadth of different experiences.

The analysis process was conducted in two steps to develop the focused and theorised coding of categories (Charmaz, 2014). First, analysis of explored individual player experiences was utilised in detecting and structuring preliminary categories. Second, an analysis and comparison of individual reflections of the selected coaches, observational logs, and team reports of coaching staff meetings were conducted. The initial researcher reflexive log together with the research log of observations during the action process (Smith and McGannon, 2018), were supplemented with observational memos and theoretical notes (Tufford and Newman, 2010) during the data analyses process. The outcome of these analyses and debriefing reflections is presented as proposed categories in a construct of elements suggested bridging shared understanding and interaction of activities handling challenging situations developing team resilience.

### Findings and Discussion

A shared understanding of challenging situations in this study built on the presumptions that such a deliberate appropriation is founded in the “we” (Salice et al., 2019; Szanto and Krueger, 2019) of the team based on dialogue between team members was developed. The consequence of such an interpretation is the assumption of team members engaged in the activity of shared understanding contributing to the sensemaking of the situation with their past experiences and future coordinated actions in a dialogic (Rommetveit, 2008) manner.

The two main categories, *role expectations*, and interpersonal *linking strategies* were found to facilitate coordinating actions between members of the team. First, different role expectations which have an impact on the appropriation and coordinating activities are presented. Then, three different intraspecific strategies which link interpersonally coordinated actions are presented. Finally, by putting the categories together, a model of interaction is suggested to facilitate the appropriation and development of shared understanding in challenging situations.

#### Role Expectations

Connecting experiences from the past with the present is important both from an individual and team perspective in order to develop coordinated actions in handling challenging situations. One of the coaches in this study reflected on the potential of developing challenge and support dynamics in the team by involving players, distributing responsibility, and sharing visions within the concept of the team:

I believe in challenge and demand more within the concept and using it. Take them in and talk with them…I believe we have met this player where he was and nurtured his strengths, then when the relationship is established start to challenge him. What he's good at has become a prerequisite for our play…. We talk about this team concept, I believe it's about the understanding of the whole (C1).

The reflection of the coach shows both the complexity in developing a shared understanding of challenging situations (Gucciardi et al., 2018) and the suggested need to approach team member appropriation through dialogue (Rommetveit, 2008) activities. First, the coach emphasised meeting players where they are with their abilities. In this, he suggests building on the characteristics and strengths of the player. Second, he wants to challenge players within the concept of the team when the relationship is established.

The example of the contribution of this particular player to the development of the team and the general expectation of the coach when it comes to challenging players within the identity of the team represents the ongoing balance of being an individual player and a member of the team, which seems to follow the different elements found within role expectations in this study.

The same coach (C1) also stated: “*To sit down with a particular player, you must ‘see the big picture,’ see what will happen and that this is a challenge in our environment.”* This statement encapsulates another aspect of developing a shared understanding of challenging situations. The understanding of different players of the “team's big picture” is individual and needs to be clarified as part of developing a shared understanding.

To capture different multiple facets of interpretation and make individuals connect with a shared image (“big picture”) of team actions is a complex mixture of connecting the expectations (past experiences and future predictions) of the players (and coaches) with the present identity of the team. Involving the experiences of players which had been established through the institutional and organisational guidelines, norms, and practise of the team seems essential because it affects the appropriation of the shared understanding of coordinative actions for handling difficult situations. At the end of the season one player in the shared leadership group reflected on the achievements of group activities:

I think we have come far with the constellation of us in the team unit. We have a good picture of how we are supposed to do things and agree on it. To bring others in and align their thinking with the way we think is an important next step in enhancing the relationships between the team units in different aspects of the play (STLGP2).

This was a player whose role was exposed to on-pitch challenges handling crunch-time situations on a weekly basis during the season. The statement of the player of having a “good picture” of how they were supposed to do things and most importantly, agree on how to do it, invites exploration of coordinative elements, which is in line with the findings of Morgan et al. (2019) when they found similar statements like “knew what to do” and “nothing was a surprise”—indicating that team members are on the same wavelength in developing team resilience.

The alignment of thinking to enhance relations between team units in different aspects of the play is recognised by the players in this study. There is a need for clear expectations within and between roles (Gucciardi et al., 2018) and the expectations might be both articulated and knowledge silently developed by the interaction of team members. This indicates that the relationship is influenced by personal preferences and that getting to know team members (Giske et al., 2018) through role clarity is beneficial for developing shared understanding between team members.

Establishing such *role clarity* based on the role expectations is another aspect of developing a common understanding of a situation. Creating dialogues to discuss challenging situations through strengthening communication channels (Kegelaers et al., 2020), is shown to capture more implicit elements in coordinating actions. Talat and Riaz (2020) suggest utilising team member bricolage as a combination of resources at hand in the team in order to develop the sensemaking of a team. One player elaborates his view on utilising small-sized group structures, explaining his belief in discussion and collaboration while debriefing and planning actions through video-analysis reflections of handling challenging situations:

When we sit together you get five or six different views and you could agree on different solutions on aspects that might be the case if you just think on your own. Thinking individually, you might be locked into a fixed mindset of what to do, but if you get inputs, it's easier to bring in the ideas, and you collaborate better out on the pitch when you agree on things together instead of just sitting on it yourself (P4).

Different views might be beneficial for developing the sensemaking managing and coordinating the effort of the team by explaining and anticipating situations (Talat and Riaz, 2020), but as the player reflection shows, shared understanding needs agreement. In line with the suggestions of Morgan et al. (2019), enablers such as shared vision, encouragement of honest feedback, and distributed responsibility are found to cultivate such mutual trust during difficult periods. One player mentions the importance of knowing each other in order to develop common understandings while reflecting on challenges he was exposed to during the season:

I felt you pushed me pretty good, and everything was new to me. Next year we probably could push much harder, but I think this year was good. Maybe trust me even more as a player on what I need of exposure. This demands a bit; you need to know each other to make this happen (P7).

The need coaches have for building trust in the capabilities of a player in order to facilitate his exposure to challenges, highlighted by the player, is in line with earlier findings. Inherent to briefings and debriefing discussions, Morgan et al. (2019) promote honest feedback to build trust among members, connecting it to coordinative activities in different situations over time. Strengthening communication in line with the work of Kegelaers et al. (2020) makes the team less vulnerable (Decroos et al., 2017) and reduces the likelihood of collective collapse (Wergin et al., 2018) when facing challenging situations. As the player suggests, getting to know and trust each other demands a bit, and it takes time to make it happen. Such individual and team collaboration of the development over time seems to be founded in different goal processes. Which one player summarised in his reflections:

*I think I've just grown into the role of demanding things from my teammates. I want us to perform as a team getting as many points as possible, which is what we all work for, both players and coaches. We have had short-term goals and long-term goals. In the beginning, I was struggling, now I dare to push the limits and dare to be more offensive in my head* (P8).

The deliberate use of the goal-setting approach reported by this player in order to achieve individual and team-related outcomes is in line with the emphasis of Morgan et al. (2019) on building team-regulatory systems based on ownership and responsibility among the team members.

It also shows a need to be aware of the processes of adaptivity and flexibility interacting with challenge and support in the development of team resilience. It confirms the attention of LePrince et al. (2018) to the collective efforts and cooperative actions that have to be developed by teammates in order to withstand team-specific stressors. The reflections of players emphasise different elements of perceived social support when experiencing role challenges in the team. One interpretation might highlight the ability of this player to practise cognitive restructuring and information sharing (Bowers et al., 2017) and being a player utilising the complementary expertise of the whole coaching team, as recommended by Morgan et al. (2019). Players explicitly endorse the need for having support staff available when the going gets rough:

To have someone from both parties to talk to, share ideas and thoughts, and get a perspective on things. I know from my experience it is good having someone keeping the family outside maybe. Often it is just a small blowout and it's all there is, without making a fuss about small details (P8).

This is an example of the differentiated need for support in different roles. Another player aligned his goal-setting and self-regulated learning processes with the vision of the team by matching his own resources with role expectations in the concept of play, taking advantage of resources in the coaching staff handling a challenging situation of being deselected over time during pre-season. His reflection pinpoints the need for coordinated support:

I experienced being pushed by the coaches, and the two main tasks I've received feedback on have been aligned with my personal goals. Which I've really been working on. It has made me succeed time after time and this made me more relaxed. Without being conscious of it this has automatically made me more relaxed on the pitch (P3).

The facilitation of working communication (both verbal and non-verbal) helps team members achieve “being on the same wavelength” as suggested by Morgan et al. (2019). The strengthening of formal communication channels connects players and coaches through interaction. The finding in this study is in line with the description of Kegelaers (2019). Not just to be told as a player what to do, but to reflect on thoughts and feelings, especially when things get a bit out of hand before they are handled. As one of the players reflects in evaluating his journey at the end of the season:

I've had coaches challenge me really hard, and coaches supporting me. Nothing beats this combination. I think coaches must keep on pushing players, but as I said there is a limit and I think we have balanced it. Believe in ourselves, have people around you that dare to push you and if it goes wrong, keep your head up and keep learning from situations and experiences in life, then you can reach for the stars (P8).

In the reflection of the experience of the player during the season, he emphasised the need to rely on each other within the team. He also made a critical remark about what needs to be improved within the team in the future. It is something that consciously should be reflected on in initiating strategic interactional engagements and developing team resilience through role expectation, role clarification, clear goal-setting processes, and flexible support in the team. In this study, these elements are found to have an impact on the shared understanding of challenging situations in the team.

#### Intrapersonal Linking Strategies

In order for the team members to be “on the same wavelength” (Morgan et al., 2019) it is important to follow individual stages of development and build communication channels (Kegelaers et al., 2020). This contributes to making the team less vulnerable (Decroos et al., 2017) and avoiding sudden team collapse (Wergin et al., 2018). Coming to the situation with different baggage and backgrounds, team member knowledge (Giske et al., 2018) is shown to be essential in this study, in order to be able to bridge shared understanding of coordinated handling of challenging situations. By studying dyadic relationships as recommended by LePrince et al. (2018), it is possible to search for linking strategies of team member knowledge through needed coordinating activities in a professional football context (Giske et al., 2018).

One player addresses the observation of both players and coaches interpreting situations through statements like “*I feel the biggest challenge is related to my own expectations of goals I've pictured compared to the reality* (P2),” and “*it's like I almost care too much about how things turn out* (P2).” To be able to adjust such perceptions handling task-specific challenging situations resourcefully together is essential, as suggested by Gucciardi et al. (2018). Another player addresses the aspect of handling different individuals;

Yeah, you could push more but it also depends on the personality of the player. Because some players, if you get on their backs too much they will give up, they will get tired. If you push me, I will not get tired. They need me, so they trust me I think, and it will give me the confidence to be ready for every game until the last one (P6).

Accessing information such as the motivational strategy of a player is an environmental resource and, as mentioned above, connecting with the personality of a player might be experienced. Such player and coach experiences of the activities of the team could provide insights into relational elements nurturing potential team resources. As recognised, team member knowledge is present where players understand each other by recognising strengths and weaknesses and different preferences (Giske et al., 2018).

Intrapersonal strategies as a bridging resource then become of interpersonal interest when modelled as team member knowledge (Giske et al., 2018) and utilised as working communication (Morgan et al., 2017). Different situational events with individual reflections of experienced interactional activities suggest that three strategies should be investigated.

#### Motivation as Interpersonal Links

First, the motivational element shown in the interviews when players reflected on handling challenging situations was such a strategy. One player explicitly mentioned motivation as a challenge on its own when interacting with coaches and team members;

It's challenging to stay motivated when I feel there is an endless road ahead of me being where I want to be. I feel the biggest challenge is related to the expectations I have for myself comparing the goals I've set for myself, how I've pictured it and how it is (P2).

By experiencing the lack of trust of being left out of the team, this player worked hard by refocusing his approach in practise. It also became part of appropriating different shared situations. The reflections of this player exemplify the need for cognitive, affective, and behavioural restructuring shown by Bowers et al. (2017). In relation to role expectations (Gucciardi et al., 2018) and member knowledge (Giske et al., 2018) in the team, these processes need to be part of the support within the team, which indicates that the motivational strategy of players might be explored as part of the shared information between team members and part of the team protocols (Morgan et al., 2019) coordinating responses of challenging situations.

#### Learning as Interpersonal Links

Another strategic element in developing knowledge of team members (Giske et al., 2018) is learning strategy. The shared team leadership group reflected on activities and processes at the end of the season. One of them emphasised team learning as an element of progression, emphasising the need for different group structures:

The group has taken steps on all aspects compared to last season. There is more to achieve but I think things have been done in a way that we have taken small steps and continuously improve. We should keep going with more meetings based on stratification but with different constellations of relationships to get players to dare to share their opinions (STLGP1).

The stance of the leadership group to have even more group meetings with different constellations and content is reinforced by the reflections of another player:

Every day I learn. We have worked a lot on the box play, like last year we were talking about the triangle inside the box. There is always something new coming into it and I have learned a lot. We're not here to assign blame so that's why I always listen and try to do what we have said and if it doesn't work, I will talk and try to find the issue and find something we can agree on (P6).

A willingness to learn is shown and he shares information on how to optimise the learning process for him by listening to others and discussing the opportunities offered by the solutions. Another player did reflect on the combination of different approaches utilised in the team:

I think we have had a good combination of discussing on the pitch and with the video analyses. It's important to have a dialogue both on and off the pitch to try it out. In the process we did it step by step, didn't skip anything. If you got suggestions from others, then it's easier to find solutions. You got a better interconnection out on the pitch when you agree on things together instead of sitting with it all by yourself (P4).

The reflection of the player supports both visual and verbal instructions to facilitate learning in the cognitive, affective, and behavioural response to actions based on interconnecting through a “we-narrative” based on plural Self-Awareness suggested by Salice et al. (2019) and collective intentions, goals, and norms suggested by Szanto and Krueger (2019) in the expert musician environment. This implies a potential of supporting cognitive restructuring and information sharing in the responses (Bowers et al., 2017) for handling challenging situations. It underpins the importance of facilitating working communication between team members, recognised by Morgan et al. (2019). The approach includes knowing different team members (Giske et al., 2018), learning strategies in order to facilitate the quality of honest feedback (Morgan et al. 2019, and building trust to enhance relationships within the group Morgan et al. 2019).

In such video analysis, member information comes from small-sized groups and one-on-one meetings facilitated by team action training for handling challenging situations. It emphasises the need for modelling team member interaction by including the learning strategies of members as coaching elements bridging shared understanding of information in briefing and debriefing situations.

#### Individual and Team Decisions as Interpersonal Links

A third strategic bridging element coming from the reflection of both the players and coaches was individual and team decision strategies for handling challenges. The importance of this was emphasised by the shared leadership in their evaluation of activities during the season:

I think sometimes it is important to design the necessary constellations to have different stratification. Not always but sometimes. I also think it might be focused on a few scenarios. I think it's really important for us, but as I said we have now come so far in our play during matches. The relationships through signs and decisions are massively important (STLGP2).

Reading signs and making collective decisions through shared understanding seems important and it is linked to team performance under stress, which is in line with team resilience development processes (Gucciardi et al., 2018). It allows members to coordinate themselves through working communication (Morgan et al., 2019), and in this study, supporting the handling of crunch-time situations for developing the team is addressed as in this situation when coaches reflected on challenges and the potential improvement of one player:

He did take that fight at the match yesterday and I think he has grown. He has given us a new dimension regarding the balance in our play with the understanding of the play within his role and the positioning play. Of course, this needs to be developed as well, but also the choices with the ball. I think he might be struggling a little bit with seeing the whole pitch well enough (C1).

The explicit need to improve choices with the ball and connect with the ability to see the pitch is mentioned by the coach and part of the development process in the team.

Another coach reflected on a different player handling challenging situations, and talked about the ability to percept in advance, to both anticipate and utilise the information to dare to challenge his own choices in ways the concept of play of the team expects him to do. This also suggests that decision-making strategies are supposed to be part of team protocols. Structuring decision-making information helps develop a shared understanding within the team. Then the team needs to facilitate developing interaction practise that initiates and supports collaboration in line with the findings of Salice et al. (2019) in the interactions of world-class musicians. The sense of shared agency is based on triggers of cognitive and affective processes. A “we-narrative” needs to be developed by “motor-resonance,” “explicit coordination,” and “interkinesthetic affectivity” (Salice et al., 2019).

By gathering and sharing team member knowledge, coaches can facilitate a shared understanding of challenging situations. The results presented through the categories of role expectations and intrapersonal linking strategies (motivation, learning, and decision-making) are found to support the appropriation process of developing a shared understanding of information when handling challenging situations.

### Interactional Model

**Figure 1:**
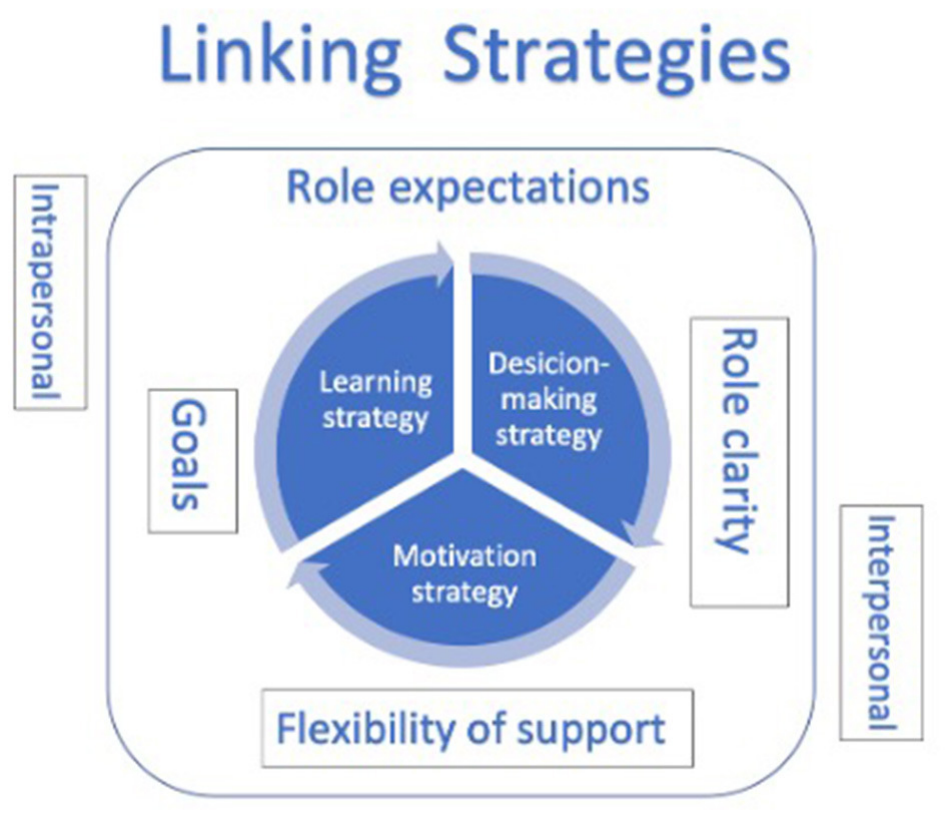
Strategic elements which contribute to facilitating shared understanding between players on and off the pitch in coordinating actions to handle challenging situations as a team.

The results are illustrated in a model of interaction (Figure 1). This model suggests key strategic elements that bridge shared member appropriation of information to develop a common understanding of challenging situations. It helps coaches and players strengthen role interactions between team members handling challenges with coordinated actions. There is a need to bridge such shared information between players developing team resilience in professional football.

Being on “the same wavelength” in order to develop a shared understanding by exchanging information through both verbal and non-verbal channels, between two or more team members, is recognised as a significant part of team-coordination mechanisms (Morgan et al., 2019). Therefore, the model suggests structuring the working communication through role expectations and linking strategies, which in turn strengthens the communication channels within the team (Kegelaers et al., 2020). By combining role expectations with intrapersonal strategies, this study contributes a new understanding of the knowledge of team members in developing a context-specific team-regulatory system: a structure that holds team members responsible for team communication and cooperation while handling stressors. Bridging elements is presented as a model of interaction (Figure 1) to guide coordinative activities within a team.

The significant elements in the model represent a way to develop and strengthen constructs of shared understanding of challenging situations while building a communicational bridge in order to facilitate coordinative actions within the team.

Coaches gathering intrapersonal information of team member knowledge connected to the role expectations of a member as a proactive activity to facilitate coordinative activities is an asset when exposed to challenges. By clarifying the roles of members, utilising goal processes, and flexible support within the group, the information helps in structuring a shared understanding of a situation. Together with the knowledge of how individuals become motivated, learn and make decisions, it helps to structure the interpersonal connexion of communication between team members. This information presented in a proactive and dialogic way is found to enhance collaboration and common trust.

The interactional model suggested to facilitating communication and nurturing a shared understanding of challenging situations functions as a bridge over troubled water when interactional dynamics are invested and facilitated in advance. Processes of such interaction need to be calibrated and adjusted continuously to strengthen links between players and the coaching staff nurturing coordinative actions through individual experiences within the team. There is a need to further explore the interactional model as part of facilitating team resilience resources in different context-specific environments.

### Practitioner Exploration and Implications

As part of the activities in the study, players were expected to utilise individual and collective resources, to handle adverse events in the team resilience development processes (Gucciardi et al., 2018; Morgan et al., 2019; Kegelaers et al., 2020). The model of interaction (Figure 1) was developed through collaboration between players and players and coaches. They collaborated on plan and debriefing meetings, coordinated actions, and supportive coaching.

The strategic elements in the model started off as random elements of interaction between team members as part of the coaching to be able to resist, bounce back or grow from challenges and setbacks by facilitating team resources. During the development process, the consciousness of sharing the knowledge of members has grown, and baseline states and behaviours were inspected, discussed, and adjusted. The model emerged as a result of the opportunity to collect, analyse and reflect upon the diversity of experiences in the development processes of the team during the season.

As part of the study, a preselected group of players was followed in the environment, and expectations of the team were made through role clarity and goal-setting processes. Player strategies of learning, motivation, and decision-making were mapped and enhanced in the working communication dealing with adverse situations during briefing and debriefing of the play. Some players who participated in the under 21 Norwegian national teams and for a limited period were challenged and supported. The strategic elements of interaction to develop a shared understanding of information were utilised in this environment as well. This supplemented the study with information coming from an international level.

The evaluation of players when it comes to team processes aligns with the shared leadership reflections of the group on the actions in the study. Based on their experience, they suggested having even more framed discussions between different units in the team in order to get to know each other better, percept and think more similarly within the identity of the team by sharing information for constructing a common understanding. They emphasised the benefits of demanding more as well when everybody knows exactly what you mean at once and do it out on the pitch. It seems to enhance the focus and players dare even more to challenge “out there” in practise. It shows the need to enhance the processes of building relationships by bridging shared information within the teams. Coming from the reflections of coaches in this study, it also shows that the structure of such work has room for improvement.

The shared leadership group also suggested if someone hides, the support needs to be there with a tuned mindset in the group when they step out on the pitch. It is a complex process with players who continuously interact with a communicational network of influence. Players, coaching, and support staff all participate in the complex development of team resilience. This is why we need to make sure that the interpretation of the team identification and the individual contribution to reinforcing the team as a unit is anchored in the team through coordinated expectations and by linking strategies as structural elements within the environment itself.

## Conclusion

This study followed a professional football team in their exploratory journey to avoid relegation from the Norwegian top league (Eliteserien). Elements that nurtured a shared understanding between participants were explored while handling challenging situations in the team. The experiences of players and coaches of challenges and support during the season were investigated and a coordinative model is presented. The proposed model facilitates interactions through communicational markers to be collected and worked on within the environment.

The one-dimensional presentation of team resilience development processes as inclusive and positive for the team might be a limitation in the study. In a “selfless” sports culture, it is important that individuals do not act at the expense of their own health and well-being. A more multidimensional approach that encapsulates such team processes to also include dysfunctional aspects with individual nuances is beneficial for studying the whole picture from the point of view of a practitioner.

Data collected in this study represent a sports-specific environment and the research is planned, activated, and reflected upon by a member of the coaching staff. It is beneficial because the close connexion helps catch nuances in the environment for data gathering. But it might also be a limitation of the study with information being biassed through pre-assumptions, even though engagement of a critical friend encouraged the context-specific reflexivity. Future projects might engage several environments, groups, and sports contexts as well as different methodical approaches.

This study conducted a team-level investigation, which was assessed through a variety of conditions and situations, as recommended, by Gucciardi et al. (2018) within different contexts generally and by Morgan et al. (2017) within team sports specifically. Exploring the interactive experiences of team members during a competitive sports season might be recognised as a strength in this study as it is in line with suggestions coming from both Morgan et al. (2019) and Kegelaers (2019).

Moreover, the current study provides accessing real-world activities and practise through the close collaboration between coaches with the researcher being a regular member of the coaching staff. In line with the suggestions of Morgan et al. (2019), it offers deeper insights into strategies, enablers, and actions. The design of a season-long study provided opportunities to collect multiple sources of information in order to investigate nuances of interactional elements within the team resilience development process. This builds on Kegelaers (2019) suggestions of developing strong communication channels and enhancing the relationship processes between players and coaches.

The presented model illustrates how it is possible to build a bridge over troubled water by helping team members to be on “the same wavelength” in handling challenging situations in order to develop team resilience in professional football.

## Data Availability Statement

The raw data supporting the conclusions of this article will be made available by the authors, without undue reservation.

## Ethics Statement

The research project was approved by the Norwegian Centre for Research Data (NSD). Members were informed of the study and the researcher's mixed role. Prior to the study, informed consent was given.

## Author Contributions

The author confirms being the sole contributor of this work and has approved it for publication.

## Conflict of Interest

The author declares that the research was conducted in the absence of any commercial or financial relationships that could be construed as a potential conflict of interest.

## Publisher's Note

All claims expressed in this article are solely those of the authors and do not necessarily represent those of their affiliated organizations, or those of the publisher, the editors and the reviewers. Any product that may be evaluated in this article, or claim that may be made by its manufacturer, is not guaranteed or endorsed by the publisher.
